# Caring Again: Parent Caregivers for Their Wounded Adult Children Veterans

**DOI:** 10.1093/geroni/igab046.1381

**Published:** 2021-12-17

**Authors:** Linda Nichols, Jeffrey Zuber, Robert Burns, Jennifer Martindale-Adams

**Affiliations:** 1 VA Medical Center 11H, Memphis, Tennessee, United States; 2 University of Tennessee Health Science Center, Memphis, Tennessee, United States; 3 University of Tennessee Health Science Center, University of Tennessee Health Science Center, Tennessee, United States

## Abstract

With military personnel in Iraq and Afghanistan surviving what were previously fatal injuries, there is ongoing discussion about how to provide care for them and support their families. Parents frequently provide care for their unmarried, injured adult children, especially those returning with polytraumatic injuries, PTSD, or Traumatic Brain Injury (TBI). Parents (n=160) of combat injured adult children who participated in a DoD-funded behavioral intervention study are described. Parents were mainly mothers, average age 60.2 years, with ages ranging from 45 to 79. The veterans had functional limitations, and only 9.2% were employed. Parents, on average, had been caregivers for 6.6 years and daily spent 7.7 hours providing care and 17.2 hours on duty, primarily focused on supervision and daily life management rather than physical care. Average caregiver burden score approached high and was related to veteran TBI diagnosis, aggressive behavior toward others, and functional limitations. Few parents (22.7%) worked full-time; 85.3% had decreased personal spending, 84.0% dipped into personal savings, and 58.9% reduced retirement saving. These findings are similar to those of aging parent caregivers of adult children with serious mental illness or developmental disabilities in amount of care provided to their adult children, their level of burden, financial and career cost to themselves, and concern about their future and their children’s future. As these parents and their adult children age, providing care and resources will present greater challenges for them, for the military and veteran care systems they rely on for support, and for society.

